# Physical Activity and Academic Achievement: An Umbrella Review

**DOI:** 10.3390/ijerph17165972

**Published:** 2020-08-17

**Authors:** Ana Barbosa, Stephen Whiting, Philippa Simmonds, Rodrigo Scotini Moreno, Romeu Mendes, João Breda

**Affiliations:** 1 EPIUnit—Instituto de Saúde Pública, Universidade do Porto, 4050-600 Porto, Portugal; ana.barbosa.02@gmail.com (A.B.); whitings@who.int (S.W.); romeuduartemendes@gmail.com (R.M.); 2 European Office for the Prevention and Control of Noncommunicable Diseases, World Health Organization Regional Office for Europe, 125009 Moscow, Russia; 3 Nutrition, Physical Activity and Obesity Programme, World Health Organization Regional Office for Europe, 2100 Copenhagen, Denmark; pippa_s@live.co.uk (P.S.); bni.rodrigo@gmail.com (R.S.M.)

**Keywords:** physical activity, exercise, academic achievement, children, adolescents, school-age

## Abstract

Background: This umbrella review aimed to summarise the evidence presented in systematic reviews and meta-analyses regarding the effect of physical activity on academic achievement of school-age children and adolescents. Methods: A comprehensive electronic search for relevant systematic reviews and meta-analyses were performed in Pubmed, Cochrane Library, Web of Science, Scopus, and Latin American and Caribbean of Health Sciences Information System, and reference lists of the included studies, from inception to May 2020. Studies were included if they were systematic reviews or meta-analyses, included school-age children or adolescents, the intervention included physical activity, and the outcome was the academic achievement. Two independent authors screened the text of potentially eligible studies and assessed the methodological quality of the studies using the AMSTAR 2 tool. Results: Forty-one systematic reviews and meta-analyses that examined the effects of physical activity on children and adolescents’ academic achievement were identified. Overall, the systematic reviews reported small positive or mixed associations between physical activity and academic achievement. From meta-analyses, it was observed that physical activity had null or small-to-medium positive effects on academic achievement. Chronic physical activity showed a medium positive effect on academic achievement, and acute physical activity did not demonstrate benefits. Conclusions: Physical activity seems not to be detrimental to school-age children and adolescents’ academic achievement, and may, in fact, be beneficial.

## 1. Introduction

It is well documented that physical activity (PA) has beneficial effects for both physical and mental health [1]. If sustainable development goals are to be met, it is essential that PA levels increase among all age groups but particularly among children and adolescents. However, physical inactivity is growing significantly for young people, in part due to the rise in school-related sedentary behaviours [2]. 

The World Health Organisation (WHO) recommends children and adolescents aged 5–17 years to achieve a minimum of 60 min of moderate-to-vigorous PA (MVPA) per day [3]. The accomplishment of recommended levels of PA is crucial for the development of cognitive, motor, and social skills, as well as good musculoskeletal health [4]. However, in 2016, it was estimated that only 34% of young Europeans met these recommendations [4]. At school, a study found that European pre-adolescent children (aged 10–12 years) spent an average of 65% of their school time in sedentary activities, while they spent only 16 min per school day (5%) engaged in MVPA [5]. Investing in school policies that encourage PA would align with the WHO’s Global Action Plan for Physical Activity 2018–2030 [6], and the Physical Activity Strategy for the WHO European Region 2016–2025 [4], both of which call for countries to take action to increase the PA of children and adolescents. 

For this population group, schools are an ideal setting to promote population health by providing more opportunities to be physically active at school [7]. However, the school environment can promote a sedentary lifestyle by compelling students to sit still for long periods in the classroom (around 3.5 h, corresponding to approximately 70% of class time being sedentary) during lessons and other study activities [8]. Moreover, physical education (PE) has not yet been prioritised in many countries due to the prominence of other subjects, such as mathematics, languages, and sciences in which achievement is assessed by standardised testing methods [9]. The time dedicated to PE and active play is increasingly eroded in favour of sedentary studies, a practice that may not, in fact, be associated with higher test scores [10]. Aside from academic achievement (AA), PA has a potential range of health benefits, including reduced risk of cardiovascular and metabolic disease and improved bone health [3]. Taking a holistic view, promoting PA in schools may also contribute to the achievement of the Sustainable Development Goals [11], in particular, Goals 3 (good health and wellbeing), 4 (quality education), 5 (gender equity), 10 (reduced inequalities), 11 (sustainable cities and communities), and 13 (climate action).

While there is no clear evidence that increased PA during childhood is associated with increased PA as an adult [12], it has been shown that higher AA is associated with higher socioeconomic status (SES) as an adult, independently of SES at birth [13]. SES is a key determinant of health throughout the life course, and therefore, interventions that promote AA may have far-reaching economic and health-promoting effects for students. Thus, in addition to directly enhancing physical health, PA may also have an indirect effect on health if it promotes academic achievement.

It is beyond the scope of this review to discuss the concept of “academic achievement”, but it is important to note that the concept encompasses a broad range of outcomes that are influenced by cognitive, social, and environmental factors [14]. While the academic literature mainly employs grades and test results to quantify AA, more qualitative social and interpersonal outcomes of education are also vital for health and wellbeing. For the purposes of this review, “academic achievement” can be broadly defined as to what extent a student, teacher, or school has met their academic goals. In the research context, this is measured in different ways, most commonly using test scores and teacher-assigned grades [13]. AA is affected by several factors, including individual characteristics (motivation, perception of wellbeing, quality of life and parents’ support, involvement in activities, and motivation), school characteristics (human and material resources, class size, teaching, rewards, extra-curricular activities, technology, evaluation system, facilities), family support (home environment, provision of resources, the attitude of family members, education, SES, family size) [15], and community facilities (youth clubs, gyms, outdoor pursuits) [4]. 

Higher levels of PA are not only fully compatible with schools’ mandate to promote the health of their students, but, according to the literature, they are also unlikely to have adverse effects on learning [16]. Part of the effect of PA on AA is likely mediated via the brain’s executive functions [17], with PA inducing, neural growth and modification in synaptic transmission, resulting in changes in thinking, decision-making, and, particularly in the prefrontal cortex [18]. Acute PA increases physiological arousal, and thus attention and triggers the release of neurotransmitters that are thought to enhance cognitive processes. Aerobic PA that increases cardiovascular fitness is considered to improve brain function through neurogenesis and angiogenesis in areas responsible for memory and learning, as well as to promote cognition via changes such as increased oxygen saturation and glucose delivery [19]. Furthermore, there is evidence that regular PA promotes positive self-perception, emotional regulation, and cognitive functioning, all of which may be factors that contribute to enhancing AA [19,20]. In this review, PA is defined as any movement produced by the human body that involves skeletal muscle and increases energy expenditure [21].

Due to the significant implications for educational practices at the population level, a substantial body of research has been dedicated to understanding the effect that PA can have on students’ cognition, classroom behaviour, and AA [22]. However, results from previous reviews have been inconsistent, which may be due to the variety of study designs employed in this area. For example, some reviews have used moderators, such as SES, family support, age, sex, psychological variables, nutritional status, while others have not. There have also been discrepancies in the measurement of AA with both standardised and non-standardised tests being used, as well as ambiguity in the definition of AA. Finally, a range of PA interventions has been included in previous reviews, such as extra-curricular PA, PE, active classrooms, active commuting to and from school, specific modalities, and acute or chronic PA. For these reasons, definitive conclusions cannot yet be drawn [17,23,24]. 

Therefore, this review aimed to summarise the available evidence presented in systematic reviews and meta-analyses regarding the effect of PA on AA of school-age children and adolescents, and explore the effect of PA programmes or modalities on AA in specific subjects.

The relevance of this umbrella review is to address the need for evidence to inform the development of future recommendations, strategies, and policies at different levels, particularly within the education sector.

## 2. Materials and Methods 

### 2.1. Search Strategy

A comprehensive search for relevant systematic reviews was conducted in the following electronic databases: Pubmed, Cochrane Library, Web of Science, Scopus, and Latin American and Caribbean of Health Sciences Information System (LILACS), until May 2020. The full search strategy is described in Appendix A. For each selected database, we used the following search terms: (“physical activity” OR exercise OR “physical education” OR “active transport” OR “active mobility” OR walking OR cycling OR running OR training OR sport) AND ((academic OR school OR cognitive OR cognition) AND (achievement OR performance OR attainment OR function OR result)) AND (child* OR school-age OR schoolchildren OR adolescents OR youth), AND (“systematic review” OR meta-analysis). Specific search terms were modified according to the database requirements. There were no limits applied to the search and the reference lists of the included reviews were searched. The search was conducted by two authors, from inception until May 2020.

### 2.2. Study Selection

Two authors independently reviewed the search results and screened publications retrieved from databases and reference lists, according to predefined steps. First, articles were screened by the information from the title and abstract. Second, articles with potential relevance were retrieved for full-text review, and their eligibility for inclusion in the review was determined. Disagreements were resolved through discussion until consensus.

All studies that fulfilled the following eligibility criteria were included: type of studies—systematic reviews or meta-analysis; type of participants—school-age children or adolescents, i.e., six to 18 years old (studies that include data on younger or older students were not excluded if data could be interpreted for the eligible age range); type of interventions—any form of acute or chronic physical activity practice and; type of outcome—academic achievement. 

Studies were excluded according to study type (editorials, comments, case reports, guidelines, conference abstracts, other reviews); studies which focused exclusively on participants with cognitive disabilities (e.g., autism, attention deficit hyperactivity disorder); studies without any form of PA; studies without measurement and quantification of the outcomes of interest; and studies without available full-text.

For this review, AA refers specifically to grades and standardised test results, and only reviews that reported this outcome were included. “Academic performance” (AP) is sometimes used as a synonym for AA. At the same time, it describes these outcomes plus other measures, such as attendance, classroom behaviour, time on task, or executive function. Cognitive outcomes are often included in reviews in this field, considering they play a role in academic success. We did not exclude studies that used “academic performance” or “cognitive function”, where authors defined the term clearly and reported the specific outcomes included in our definition of AA. The setting for most of the included reviews was educational institutions or the community.

### 2.3. Data Extraction

Each selected review was independently evaluated by two authors to extract information regarding the study design, objectives, participants (type and age), number and type of included studies, PA intervention and outcome measures, the setting of intervention, subject-specific effects, the overall effect of PA on AA, and the effect sizes from the meta-analyses. If there were discrepancies in data extraction, the authors discussed until there was consensus.

### 2.4. Methodological Quality

Two independent investigators evaluated the methodological quality of each eligible systematic review, using the AMSTAR 2 tool [25]. This instrument has 16 items and enables appraisal of systematic reviews of randomised and non-randomised studies of healthcare interventions. Each study is rated according to critical domains that can affect the validity and the conclusion of the review. The critical domains considered for this review were protocol registration before the commencement of the review (item 2); adequacy of the literature search (item 4); justification for excluding individual studies (item 7); risk of bias from individual studies being included in the review (item 9); appropriateness of meta-analytical methods (item 11); consideration of the risk of bias when interpreting the results of the review (item 13); and assessment of the presence and likely impact of publication bias (item 15).

The studies were rated as ’high-quality’ if no or only one non-critical weakness was present; ´moderate-quality´ if there was more than one non-critical weakness; ´low-quality´ if there was only one critical flaw with or without non-critical weaknesses; and ´critically low-quality’ if there was more than one critical flaw with or without non-critical weaknesses. Any disagreements in the classification were resolved by discussion.

## 3. Results

### 3.1. Study Selection

**Figure 1:**
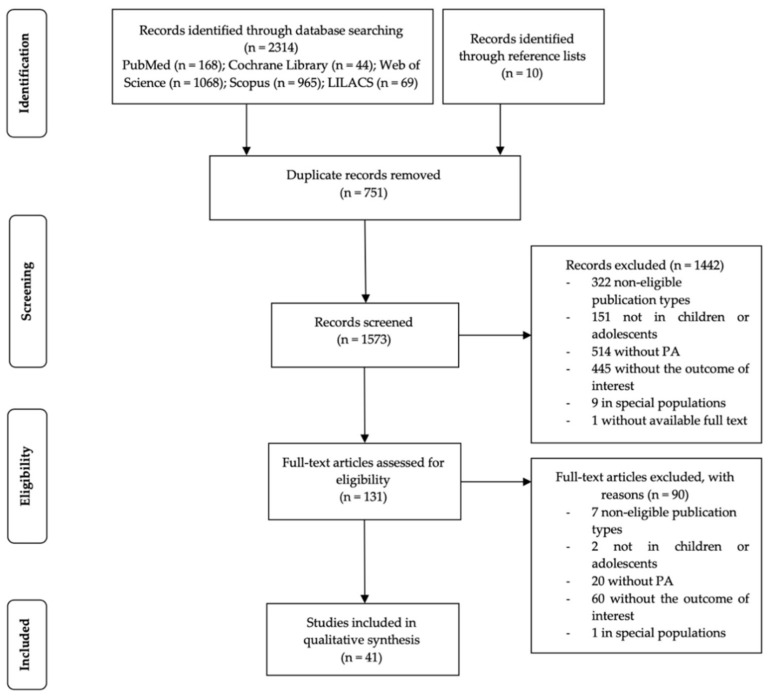
Flow diagram. LILACS, Latin American and Caribbean of Health Sciences Information System; PA, Physical Activity.

A total of 2314 references were identified in the initial search in electronic databases, and an additional ten references were identified through reference lists. After the duplicated studies were removed (*n* = 751), 1573 studies remained. After screening for the title and abstract, 1442 papers were excluded, and 131 studies were eligible for full-text reading, from which 90 were removed. Thus, we included 41 studies for qualitative synthesis (Figure 1).

### 3.2. Study Characteristics

The characteristics of the studies included in this review are shown in Table 1. Briefly, studies ranged from 2003 to 2020; all studies were published in English, except the one that was published in the Spanish language [26]. All studies were systematic reviews, and 15 provided meta-analyses [23,24,27,28,29,30,31,32,33,34,35,36,37,38,39]. The study design of the original reviews included only randomised controlled trials (RCT) or cluster RCT [29,35,40,41], and mixed designs (RCT, cluster RCT, cross-over designs, quasi-experimental studies, cross-sectional, and cohort studies) [9,17,23,24,26,27,28,30,31,32,33,34,36,37,38,39,42,43,44,45,46,47,48,49,50,51,52,53,54,55,56,57,58,59,60,61,62].

Regarding the participants, the studies included children [17,32,33,38,57,62], adolescents [26,30,47,48,52,56], or both populations [9,23,24,27,28,29,31,34,35,36,37,39,40,41,42,43,44,45,46,49,50,51,53,55,58,59,60,61], with the age ranging from one month to 21 years old. 

Concerning the type of PA intervention, two studies focused on active commuting to and from school [23,24]; one studied a yoga intervention [29] and another in school gardening participation [57]. The remaining reviews focused on active breaks and physically active lessons [31,32,35,38,46,50,58,62], and on increasing PA through a variety of forms, including PE lessons, aerobic PA or extra-curricular PA) [9,17,26,27,28,30,31,33,34,36,37,39,40,41,42,43,44,45,46,47,48,49,50,51,52,53,54,55,56,58,59,60,61]. Almost all the interventions were delivered in the school setting, including classrooms or playgrounds, and one study also reported interventions in the community context [9,51].

Studies reported distinct definitions and instruments to assess AA, such as school grades, grade point average, standardised test scores, and subject-specific test scores (e.g., mathematics, reading, spelling).

### 3.3. Study Outcomes

#### 3.3.1. Overall Academic Achievement Reported in Systematic Reviews

The effects of PA on AA are described in detail in Table 2. In general, there were small positive effects of different types of PA on AA [27,30,31,32,37,39,42,44,46,49,50,55,57,61] or mixed (small positive and null) effects [9,17,26,29,33,35,41,43,47,48,51,52,53,56,58,59]. 

Regarding the effects of PA on specific subjects, the included reviews evaluated reading, language, science, spelling, geography and mathematics. Mathematics was the subject where positive associations were found with more consistency [27,31,33,36,39,52,61]. 

#### 3.3.2. Overall Academic Achievement Reported in the Meta-Analyses

Thirteen studies performed meta-analyses and provided effect sizes for AA, which are summarised in Table 3. In brief, the overall effect of different modes of PA had null [28,32,33] or small to medium [27,30,33,35,37] effects on AA.

Considering the type of PA on overall AA, increasing the allocation of PE at school had a small [37] or medium [27,39] effects; active classrooms, compared with traditional sedentary classrooms, had null [32] or medium [35] effects. Chronic PA had a medium effect on AA; however, acute PA had a null effect on AA [33].

Regarding the effects of PA on subject-specific effects, mixed findings were observed, with studies reporting null effects or small to medium effects. The effects of PA on mathematics were null [23,24,28,33,34,35], small [39], or medium [27,31,36]. Concerning other subjects—for reading, null [28,33,34,35], small [31,39], or medium [27] effects were found; for language, null [23,28,31], medium [27], or large [35] effects were found; for science, a null effect was found in two studies [27,35]; for spelling, a null effect was found in one study [35], and another which assessed acute PA [33], as well as a null effect from chronic PA [33]; for geography, a large effect was found in one study [35].

Looking at the type of PA intervention on subject-specific achievement, active commuting to and from school was not associated with mathematics [23,24] and language [23]; increasing the allocation of PE at school had a small [39] or medium [27] effect on mathematics and reading; active classrooms, compared with traditional sedentary classrooms, had a null [35] or medium effect in mathematics [31], a null [35] or small [31] effect on reading, a null [31] or large [35] effect on language, a null effect on spelling and science [35], and a large effect on geography [35]. 

### 3.4. Methodological Quality

Table A1 shows the assessment of methodological quality for each study. Two studies were rated as ‘high-quality’, five studies with ‘moderate-quality’, 11 studies with ‘low-quality’, and 23 studies with ‘critically low-quality’. The critical domains where studies did not meet the quality requirements were the registration of the protocol before the commencement of the review (item 2, *n* = 30), the consideration of the risk of bias when interpreting the results of the review (item 13, *n* = 22), and the assessment of the risk of bias from individual studies being included in the review (item 9, *n* = 15). In non-critical domains, the majority of reviews (item 10, *n* = 39) did not report the sources of funding of individual studies (item 10), did not report the complete Population, Intervention, Control group, and Outcome (PICO) components (item 1, *n* = 32), and did not explain the selection of study designs for inclusion (item 3, *n* = 28). There was total agreement between the reviewers on the methodological quality assigned to each study.

## 4. Discussion

### 4.1. Main Results

This review summarises the evidence of 41 systematic reviews and meta-analyses examining the relationship between physical activity and AA in school-aged children and adolescents. Overall, the findings suggest that PA has a null or small to medium effect on AA in school-age children and adolescents. 

Our results are consistent with the latest studies in this field [35]. However, the findings are mixed when reviews include experimental or longitudinal studies. Mathematics was the subject measured more frequently, compared with other subjects, with null or small to medium effects. It has been hypothesised that PA improves executive function, which, in turn, has an impact on inhibition, working memory, and cognitive flexibility, components associated to this subject [36]. Furthermore, improving cognitive skills, such as visuospatial skills, rapid automatised naming, and memory can contribute to arithmetic learning [63,64]. Despite these considerations, more studies are needed to assess the effect of long-term PA intervention on mathematics’ performance [62], and also whether PA interventions that increase student’s enjoyment of classes can promote psychological wellbeing—another factor in academic performance [36].

The allocation of time for PE at school has a small to medium effects, and active classrooms, compared with traditional sedentary classrooms, had mixed effects. These mixed findings could, in part, be explained by methodological issues, such as the variety of type and length of PA interventions, the heterogeneity of populations included, as well as the specific definition of AA and the method of measurement utilised. In addition, a lack of moderators, including SES, family support, age, sex, psychological variables, nutritional status, may have also contributed to these diverse findings [17].

The studies which assessed chronic PA also reported small positive effects after the observational period, which is a very promising finding. To investigate this further, it is important to conduct high-quality RCTs over a longer period of time, as well as long-term and large-sample size longitudinal observational studies. 

Active commuting to and from school was not associated with improvements in mathematics [23,24] and language [23]. This may be explained by specific methodological factors, such as the different definitions employed across the primary studies and the various ways of measuring active commuting to and from school, including objective and subjective measures, the low frequency of participants who engage in active commuting to and from school, and the lack of moderators’ assessment in the studies [23]. Other environmental factors may affect the low frequency of active commuting to and from school, such as air pollution, the existence of safe sidewalks, cycle paths, and routes to school, as well as perceived neighbourhood safety [24].

The majority of the included reviews were scored as ‘low-quality’ or ‘critically low-quality’ when assessing the methodological quality. For a study being rated as ‘low’ quality, it must present one critical weakness. Nevertheless, part of these studies failed to contain an explicit statement that the review methods were established before the conduct of the review, which does not necessarily mean the study has low-quality. Furthermore, AMSTAR 2 does not intend to provide a score, so these findings must be interpreted with caution.

### 4.2. Limitations

There are several limitations that must be considered when interpreting these findings. First, the included reviews were heterogeneous in the type of interventions (single or multiple interventions), the population included (children, adolescents, or both), type of study designs, outcome measures, and lack standardised definitions for outcomes, with the terms “academic achievement” and “academic performance” not clearly defined and sometimes used interchangeably [65], which may limit clear interpretations of the results.

Second, many reviews did not include risk of bias scoring, and did not report details of participant and assessor blinding, or provided insufficient information regarding concealment of allocation to the intervention or the control group [52] and, therefore, incorporated low-quality evidence to draw their conclusions, especially the oldest publications. 

### 4.3. Implications for Practice

Innovative strategies are needed to provide adequate PA for children and adolescents. It would be beneficial to ascertain whether some types of PA, such as active breaks, could be recommended. Several European cities report less cycling and walking in the commute to and from school and policy actions to ensure that young people can actively travel to and from school, which could increase both PA and AA. Our findings may be useful to support national and local governments to design intersectoral approaches, involving the health, sports, and education sectors, that aim to improve both academic and physical development through the promotion of PA to students in and out of the school settings [4].

At the school level, teachers may need to be supported to apply innovative approaches and strategies to increase PA levels; teachers and school administrators could receive training on the well-understood beneficial effects of PA on health, and the likely beneficial effects on AA. This should include practical guidance on how to implement increased PA in schools, including how to involve students and parents in planning activities, with support from suitably qualified PE professionals to maximise its potentially beneficial effect on AA. Schools also need to have appropriate open spaces, materials and resources to provide diverse opportunities for regular PA for children and young people of all ages. Partnering with sports and community organisations can support the development of extra-curricular opportunities for physical activity [4]. 

At the community level, safe environments are needed to enable regular PA and active commuting to and from school. The availability of sports and fitness clubs/gyms, community youth clubs, such as scouts, can also increase opportunities while a range of age- and gender-specific forms of PA need to be available, especially for adolescents. Awareness of the availability of opportunities could be raised through information and communication technology, social media approaches, and community and youth organisations [4].

### 4.4. Future Research

There are a number of areas for potential future research that can be highlighted, with the most prominent being the need to establish the causality of the relationship between PA and AA. Insights may be gained from conducting high-quality RCTs with a number of different PA intervention arms in addition to a non-active control group [60]. 

Greater collaboration between exercise scientists and neuroscientists may also help to improve the quality of future research and attempts to understand more clearly the relationship between PA and AA [52].

Also, future research should incorporate standardised outcome measures and strive towards increased standardisation of PA interventions with attention to methodological rigour and consideration of relevant moderators.

While some reviews provide policy recommendations, these are often not sufficiently detailed or disseminated to the appropriate audiences [65]. Researchers may be advised to publish practical articles in association with education specialists so that their findings could actually be translated and implemented by school administrators. 

## 5. Conclusions

PA seems not to be detrimental to school-age children and adolescents’ AA, and may, in fact, be beneficial. Different types of PA appear to have different effects, with the most benefit gained from longitudinal programmes incorporating aerobic exercise. Policymakers at the national, local, and school level should be made aware of the latest evidence and encouraged to make changes accordingly. 

## Figures and Tables

**Table 1:** Summary table of included reviews.

Author, Year	Aim	Participants Type and Age (years old)	Number and Type of Included Studies	PA Assessment	AA Assessment	Setting
Sibley and Etnier, 2003 [39]	To quantitatively combine and examine the results of studies about physical activity (PA) and cognition in children	Children and adolescents 4–18	16 in SR and meta-analyses RCT, quasi-experimental, cross-sectional and correlational	Acute or chronic PA, resistance training, aerobic training, perceptual-motor, PE program	Achievement, verbal tests, math tests	NR
Strong et al., 2005 [42]	To review the effects of physical activity on health and behavioural outcomes and develop evidence-based recommendations for PA in youth	Children and adolescents 6–18	850 Quasi-experimental and cross-sectional	Addition of PE to the curriculum	Grade point average, test scores, standardised test scores	School
Murray et al., 2007 [43]	To identify and summarise the evidence about Coordinated School Health Program-related determinants of AA	Children and adolescents 6–18	17 RCT, quasi-experimental	PA or PE intervention	Grade point average, school grades, standardised test scores	School
Trudeau and Shephard, 2008 [44]	To review relationships of AP and some of its determinants to participation in school-based physical activities, including physical education (PE), free school PA and school sports	Children and adolescents 0–18	17 Quasi-experimental and cross-sectional	Increase PA or PE lessons and extra-curricular PA	Grade point average, school grades, standardised test scores	School
Fedewa and Ahn, 2011 [27]	To synthesise the research on PA and children’s cognitive outcomes and to discuss implications for educators and other stakeholders in children’s AA	Children and adolescents 3–18 years	59 Experimental, quasi-experimental, cross-sectional	Resistance, circuit training, aerobic training, PE program or perceptual-motor training	Total achievement, spelling, language, art, reading, science and math achievement, grade point average	School
Rasberry et al., 2011 [9]	To synthesise the scientific literature that has examined the association between school-based PA (including PE) and AP	Children and adolescents 5–18 years	43 Experimental, quasi-experimental, and descriptive	PE and/or PA or extra-curricular physical activities (including school sports)	Grade point average, standardised test scores, classroom test scores, other formal assessments	School and community
Singh et al., 2012 [45]	To describe the prospective relationship between PA and AP	Children and adolescents 6–18 years	14 Cohort and intervention	Self-reported athletic participation, participation in PE lessons, self-reported PA, questionnaires or recalls	School grades, cognitive tests	School
Haapala, 2012 [40]	To overview the evidence of the effect of PA interventions on cognitive ability and AA in children and adolescents	Children and adolescents 7–18	9 RCT	PA training program	Standardised test scores or academic skills	School
Lees and Hopkins, 2013 [41]	To overview research elucidating the relationship between aerobic PA and children’s cognition, AA, and psychosocial function.	Children and adolescents 0–18	8 RCT	Aerobic PA	School grades	School
Pucher, Boot and de Vries, 2013 [46]	To determine whether school health promotion interventions can enhance children’s AP	Children and adolescents 6–18	7 RCT, crossover trials, quasi-experimental with pre-post test	PE classes, daily classroom PA	School grades, standardised test scores	School
Busch et al., 2014 [47]	To systematically review the longitudinal effects of adolescents’ most prominent health-related behaviours on their AP	Adolescents 12–18	30 RCT and longitudinal	PA and team sports’ participation	School grades	School
Martin et al., 2014 [28]	To assess whether lifestyle interventions improve school achievement, cognitive function and future success in overweight or obese children and adolescents compared with standard care, waiting list control, no treatment, or attention control	Children and adolescents 3–18	6 RCT, cluster RCT and controlled clinical trials, with or without crossover design	Increase PA	Grade point average, test scores, standardised test scores	School, after-school, home
Conde and Tercedor, 2015 [26]	To summarise scientific studies published in recent years that evaluate the relationship between PA, fitness (including PE) with cognitive and AP in teenagers and colleges	Adolescents and young adults 11–22	28 Experimental, quasi-experimental, observational	Increase PE lessons, questionnaires	Grade point average, school grades, standardised test scores	School
Esteban-Cornejo et al., 2015 [48]	To perform a systematic review of the evidence on the associations between PA and cognition by differentiating between academic and cognitive performance measures	Adolescents 13–18	20 Cross-sectional, longitudinal or interventional study	Self-reported questionnaires and objective measures of PA	School grades, subject-test score	NR
Ferreira-Vorkapic et al., 2015 [29]	To systematically examine the available literature for yoga interventions exclusively in school settings, exploring the evidence of yoga-based interventions on academic, cognitive, and psychosocial benefits	Children and adolescents 5–18	9 in SR and meta-analyses RCT	Yoga	AP	School
Mura et al., 2015 [49]	To examine the effects of school-based PA interventions on AA and cognitive outcomes	Children and adolescents 3–18	31 Experimental or quasi-experimental	Increase PA, PE lessons by specialists	School grades, standardised test scores	School (classroom, schoolyard, school gym), after-school
Norris et al., 2015 [50]	To investigate the methods used in active physical lessons and their effects on PA and educational outcomes	Children and adolescents 0–18	11 RCT and non-RCT	PA lessons intervention, PA active breaks, PA active math classes	Standardised test scores	School (classroom)
Donnelly et al., 2016 [17]	To investigate if PA and physical fitness influence cognition, learning, brain structure, and brain function, and if PE and sports programs influence standardised achievement test performance and concentration/attention, among children aged 5–13	Children 5–13	73 Cross-sectional, acute, longitudinal, and intervention studies (both non-randomised and randomised)	Increase PA (physically active classroom and breaks, PE, after-school programs)	Subjects exam score and standardised test scores	School (classroom, PE), after-school
Poitras et al., 2016 [51]	To examine the relationships between objectively measured PA and health indicators in school-aged children and youth	Children and adolescents 5–17	162 All study type	Various volumes, durations, frequencies, intensities and patterns of objectively measured total PA	School grades, standardised test scores	School, community, home
Spruit et al., 2016 [30]	To conduct four multilevel meta-analyses on the effect of PA interventions on externalising problems, internalising problems, self-concept, and AA in adolescents	Adolescents 10–21	57 RCT and quasi-experimental	Participation in sports or aerobic exercise	School grades, standardised test scores	NR
Álvarez-Bueno et al., 2017 [31]	To assess the effect of PA interventions on AA and classroom behaviours in childhood and to determine the characteristics of individuals and PA programs that enhance AP	Children and adolescents 4–13	26 in SR; 11 in meta-analyses RCT, quasi-experimental and controlled pre-post studies	Active breaks, math with physically active tasks, extra-curricular PA, after-school PA or sports programs	School grades, standardised test scores	School, after-school
Li et al., 2017 [52]	To investigate whether exercise, proposed to enhance neuroplasticity and potentially cognitive function and AP may be beneficial during adolescence when important developmental changes occur	Adolescents 13–18	10 RCT, crossover trials	Chronic or acute structured exercise intervention (or both)	Arithmetic	School
Marques et al., 2017 [53]	To perform a systematic review of the evidence on the associations between PE and school-based PA, and AP	Children and adolescents 6–18	12 Cross-sectional, longitudinal or intervention studies	PE lessons or school-based PA	School grade, standardised test score, the measure of AP	School
Martin and Murtagh, 2017 [55]	To conduct a systematic review of classroom-based PA interventions that integrate academic content and assess the effectiveness of the interventions on PA, learning, facilitators of learning, and health outcomes	Children and adolescents 5–18	15 RCT, non-RCT, exploratory, pre-and post-test designs	MVPA intervention	Standardised tests, written test, and structured interviews	School (classroom)
Ruiz-Ariza et al., 2017 [56]	To investigate the association of different components of physical fitness on cognitive performance and AP in adolescents	Adolescents 13–18	21 Cross-sectional, longitudinal or intervention	PE, physical fitness, cardiovascular, aerobic, strength, flexibility, speed, agility, ability, coordination	Grade point average, standardised test scores	School
Schneider, Pharr and Bungum, 2017 [57]	To identify the impact of participating in school garden programs on fruit and vegetable preference or consumption, nutrition knowledge, PA, and standardised test scores	Children 5–13	14 Cluster RCT, quasi-experimental, mixed methods	Gardening	Subject-test score	School
Watson et al., 2017 [32]	To evaluate the impact of classroom-based PA interventions on academic-related outcomes, and to evaluate the impact of these lessons on PA levels	Children 5–12	39 in SR; 16 in meta-analyses Quasi-experimental, Cluster RCT, RCT, cross over	PA carried out during regular class time, inside or outside the classroom: active breaks, curriculum-focused active breaks, physically active lessons	School-related tasks, school grades, national standardised test scores or progress monitoring tools, and self-reported perceived academic competence	School (classroom)
Marques et al., 2018 [54]	To systematically review the evidence for a differential association between objective and self-reported PA and cardiorespiratory fitness on AA	Children and adolescents 6–18	51 cross-sectional, longitudinal and interventional study designs	Cardiorespiratory fitness, objective and self-reported PA	School grades, standardised test scores	School
Daly-Smith et al., 2018 [58]	To examine the impact of acute classroom movement break and physically active learning interventions on PA, cognition, AP and classroom behaviour	Children and adolescents 4–17	17 RCT and non-RCT	Classroom movement break and physically active learning	Subject-test score	School (classroom)
de Greeff et al., 2018 [33]	To provide a systematic review of intervention studies that investigated the effects of PA on multiple domains of executive functions, attention, and AP in preadolescent children	Children 6–12	31 in SR and meta-analyses Random assignment or matching with appropriate adjustments for any pre-test difference	Acute or chronic PA	Mathematics, spelling, and reading score	NR
Martin et al., 2018 [34]	To assess whether lifestyle interventions improve school achievement, cognitive function, and future success in children and adolescents with obesity or overweight, compared with standard care, waiting-list control, no treatment, or an attention placebo control group	Children and adolescents 3–18	18 in SR; 17 in meta-analyses RCT, cluster RCT, and quasi-randomised trials with or without crossover design	Increase PA	Grade point average, test score, standardised test scores	School, community, clinics
Bedard et al., 2019 [35]	To determine the impact of active classrooms compared to traditional sedentary classrooms on educational outcomes of school-aged children	Children and adolescents 3–18	25 in SR and meta-analyses Studies with random individuals or clusters to an intervention or control condition	Physically active school lessons’ intervention	School grades, standardised test scores	School (classroom)
Gunnell et al., 2019 [59]	To extend the generalisability of previous findings on the relationship between PA and brain health in children and youth	Children and adolescents 0–18	83 RCT	Acute or chronic PA	Subject-test score	School, after-school
Phansikar et al., 2019 [24]	To conduct a systematic review of studies done with children and adolescents, investigating the relationship between Active Commuting to School, and cognitive functioning or AA	Children and adolescents 0–18	12 in SR; 2 in meta-analyses RCT, pre-post, longitudinal, cross-sectional, and case-control	Active commuting	School grades, standardised test scores	Active commuting to and from school
Ruiz-Hermosa et al., 2019 [23]	To evaluate the link between Active Commuting to School and cognitive performance and AA in children and adolescents	Children and adolescents 4–18	12 in SR; 9 in meta-analyses Observational studies (cross-sectional or baseline assessments of cohort studies)	Active commuting	Standardised test scores, subject and classroom-test score	Active commuting to and from school
Singh et al., 2019 [60]	To summarise the current evidence on the effects of PA interventions on cognitive and AP in children, and formulate research priorities and recommendations	Children and adolescents 0–18	58 RCT and non-RCT	PA-related intervention studies	Grade point average, school grades, standardised test score	School
Sneck et al., 2019 [36]	To address if school-based PA interventions have an effect on children’s mathematics performance and identify the characteristics of PA interventions with positive effects on math performance	Children and adolescents 4–16	29 in SR; 11 in meta-analyses RCT, cluster RCT, quasi-experimental, intervention, pre-post-test design, crossover	PA before, during or after school lessons or at break time or was in the form of PE lessons	Mathematics grades, standardised test score	School
Chacón-Cuberos et al., 2020 [61]	To conduct a systematic review of the scientific literature addressing the impact of PA on AP in preadolescent young people	Children and adolescents 7–15	23 RCT, cluster RCT, quasi-experimental, cross-longitudinal	Increase PA	Standardised test scores, subject-test score	School
Dudley and Burden, 2020 [37]	To estimate the mean effect of increasing the proportion of total curriculum time allocated to PE on student learning	Children and adolescents 3–18	6 in SR and meta-analyses RCT, non-RCT, quasi-experimental, controlled pre-post and longitudinal	Increase PE	School grades, specific tests	School
Masini et al., 2020 [38]	To provide a systematic review of studies that investigated the effects of Active Break school-based interventions on PA levels, classroom behaviour, cognitive functions, and AP in primary school children	Children 6–13	22 in SR; 6 in meta-analyses RCT and observational	Active breaks: sessions of MVPA led by teachers who introduce short bursts of PA intro the academic lesson	Standardised test scores	School (classroom)
Vetter et al., 2020 [62]	To investigate the efficacy of combining math lessons with PA by reporting academic and PA outcomes in primary/elementary school children	Children 6–12 years	11 RCT, controlled trials or quasi-experimental	Physically active lessons	School grades, standardised test score, school-related tasks	School

AA, Academic Achievement; AP, Academic Performance; MVPA, Moderate-to-Vigorous Physical Activity; NR, Not Reported; PA, Physical Activity; PE, Physical Education; RCT, Randomised Controlled Trial; SR, Systematic Review.

**Table 2:** Summary of the results included systematic reviews.

Author, Year	Results	
	Overall Academic Achievement Effect	Subject-Specific Effect
Sibley and Etnier, 2003 [39]	There is a small significant relationship between PA and AA in children.	Mathematics and verbal tests showed very small effects.
Strong et al., 2005 [42]	The addition of PE to the curriculum results in small positive gains in AP. The quasi-experimental data also suggest that allocating more curricular time to programs of PA does not negatively affect AA, even when the time allocated to other subjects is reduced. Some results also suggest a relative increase in AP per unit of time. Cross-sectional observations show a positive association between PA and AP.	N/A
Murray et al., 2007 [43]	Strong evidence suggests a lack of negative effects of PE programs on AA.	A positive trend toward increased arithmetic scores, but no significant changes in reading scores when compared with controls. No significant difference was noted between groups on the mathematics or composite basic battery scores.
Trudeau and Shephard, 2008 [44]	Studies suggest that sport is more likely to benefit AA if offered in school rather than in other sports contexts. Adding time to “academic” or “curricular” subjects by taking time from PE programmes does not enhance grades and may be detrimental to health.	N/A
Fedewa and Ahn, 2011 [27]	There was a small effect between PA and AA.	Small to medium effects on mathematics, reading and language; null effect for science.
Rasberry et al., 2011 [9]	Mixed findings: PA is positively associated with AP (50.5% of the associations summarised) or has a null effect (48% of the associations).	Reported in subgroups according to the type of PA, however association or otherwise with AA not reported separately.
Singh et al., 2012 [45]	Authors found strong evidence of a significant positive relationship between PA and AP.	Positive effects on language skills, reading skills and a basic test battery; there was no significant difference for mathematics.
Haapala, 2012 [40]	N/A	In four studies, three reported a positive effect of physical training on mathematical, reading, and language skills.
Lees and Hopkins, 2013 [41]	Aerobic PA is positively associated with AA.	Non-significant effects for mathematics, language, and sciences.
Pucher, Boot and de Vries, 2013 [46]	School health promotion intervention showed that interventions targeting PA and nutrition had a small to large effects on AP, and no negative effects occurred.	Effects of different kinds of interventions varied in size and across subjects like mathematics and language. Also, positive effects were observed in subjects from which time was taken for the intervention, e.g., for additional PE.
Busch et al., 2014 [47]	In general, team sports had an overall positive impact on AA, while individual sports had no effect.	N/A
Martin et al., 2014 [28]	Null effects were found for overall AA.	Overall, null effects were found for mathematics, reading and language.
Conde and Tercedor, 2015 [26]	Regarding the association between PA and AP, when it was assessed with Grade Point Average: 11 studies found a positive association, one study with no association and one study with a negative association; when evaluated with other instruments—four studies found at least one positive outcome.	N/A
Esteban-Cornejo et al., 2015 [48]	Four studies showed no effect of PA on AP, one showed a negative association, and 11 showed a positive association. PA was more strongly associated with AA among girls than boys.	N/A
Ferreira-Vorkapic et al., 2015 [29]	Yoga had null effects on AP.	N/A
Mura et al., 2015 [49]	The majority of the studies showed positive between PA interventions and AA.	N/A
Norris et al., 2015 [50]	In the two studies assessing PA and AA, there was a significant improvement.	One study showed significant improvement across all subjects, the other only in social sciences.
Donnelly et al., 2016 [17]	Physically active lessons in general result in improvements in AA, and the addition of PE time does not; there are positive effects for acute PA on AA.	Mixed results regarding mathematics, reading and spelling.
Poitras et al., 2016 [51]	PA resulted in mixed-effects (small or null) on AA according to the study design.	N/A
Spruit et al., 2016 [30]	A significant small-to-medium mean effect size of 0.367 was found for PA interventions on AA in adolescents. Larger effects were found for grades compared to standardised achievement tests.	N/A
Álvarez-Bueno et al., 2017 [31]	AA is improved by increasing school time dedicated to PE.	Increasing curricular PE programs benefited mathematics-related skills, reading, and composite scores. Integrating PA into lessons benefited performance in mathematics only.
Li et al., 2017 [52]	AP demonstrated a significant improvement with exercise in one of two studies.	One study showed a significant beneficial effect of PA on mathematics, while another study had a small effect without statistical significance.
Marques et al., 2017 [53]	Seven articles found a positive association between PE or school-based PA and AP, four found no association, and in one there was a positive association for third-grade students and a negative association for second-grade students.	N/A
Martin and Murtagh, 2017 [55]	Studies reporting learning outcomes (including AA) showed positive effects of physically active academic lessons on AA.	N/A
Ruiz-Ariza et al., 2017 [56]	Mixed findings, with the majority of studies showing positive associations.	N/A
Schneider, Pharr and Bungum, 2017 [57]	N/A	Two studies found that students who were involved in gardening significantly increased their science test scores.
Watson et al., 2017 [32]	Classroom-based PA had a small positive effect on AA when a progress monitoring tool was used.	N/A
Marques et al., 2018 [54]	Objectively measured PA was inconsistently related to AA, although four studies of strong quality provided partial support for a positive relationship. Self-reported PA was positively associated with AA.	Mixed results between studies.
Daly-Smith et al., 2018 [58]	The study of physically active learning found no effect on AA, while the study of classroom movement break found a positive effect on AA (when breaks were at least 10 min).	Mixed results for mathematics.
de Greeff et al., 2018 [33]	No overall significant effect of acute PA on AA in children aged 6–12 years, but there was a small to moderate effect from longitudinal PA.	For acute PA, a small to moderate effect was found for spelling. No significant effects of acute PA were found for mathematics and reading. No significant effects of chronic PA were found for mathematics, reading, or spelling.
Martin et al., 2018 [34]	Compared with the usual routine, PA interventions have no beneficial effect on AA.	Null effects of PA on AA for mathematics and reading.
Bedard et al., 2019 [35]	A small positive effect of active classrooms was found, compared with traditional (sedentary) classrooms.	Positive effects found for language and geography; null effects for mathematics, reading, spelling, and science.
Gunnell et al., 2019 [59]	Results suggest that chronic and acute bouts of PA are favourable or at least not detrimental to cognitive function.	One study found moderate-intensity exercise had null (for sentence comprehension), favourable (spelling and reading), and unfavourable (math) effects on AA, compared to seated rest. Vigorous and moderate-intensity exercise groups combined had null (reading, sentence comprehension), favourable (spelling), and unfavourable (arithmetic) results on AA, compared to seated rest. Moderate-intensity exercise had null (spelling, reading, arithmetic, and sentence comprehension) results on AA, compared to vigorous exercise.
Phansikar et al., 2019 [24]	N/A	Active commuting to and from school was not significantly associated with mathematics.
Ruiz-Hermosa et al., 2019 [23]	N/A	Active commuting to and from school was not significantly associated with mathematics and language.
Singh et al., 2019 [60]	Inconclusive evidence for a beneficial effect of PA on overall AP.	Strong evidence for the beneficial effect of PA on mathematics and inconclusive evidence for language.
Sneck et al., 2019 [36]	N/A	Small to medium effects were found for mathematics.
Chacón-Cuberos et al., 2020 [61]	Most of the studies that addressed AP using the scores obtained in non-standardised tests showed PA results improved AP. Interventions with higher exercise intensity and duration led to more pronounced improvements in AP.	Two studies found that active learning and the introduction of active breaks improved AP in children with lower grades. Three studies found that the benefits of their intervention programmes were more closely associated with improvements in mathematics, with particular emphasis on arithmetic, while not finding any relationship with reading comprehension.
Dudley and Burden, 2020 [37]	Increasing the proportion of the curriculum allocated to PE had small positive effects on student learning.	N/A
Masini et al., 2020 [38]	Inconclusive evidence was found regarding the effect of school-based interventions of active breaks on AA.	N/A
Vetter et al., 2020 [62]	N/A	Four of the six studies reported statistically significant improvements in reading, but ES was small. The RCTs assessing spelling reported significantly better improvement in intervention, compared with control, with ES = 0.45; significant improvements were seen on English, reading, and spelling.

AA, Academic Achievement; AP, Academic Performance; ES: Effect Size; N/A, Not Applicable; PA, Physical Activity; PE, Physical Education; RCT, Randomised Controlled Trial.

**Table 3:** Summary of effect sizes retrieved from the meta-analyses.

Author, Year	Results			
	Overall Academic Achievement Effect		Subject-Specific Effect	
Sibley and Etnier, 2003 [39]	Overall ES: g = 0.30	●●	Mathematics: g = 0.20	●
			Reading: g = 0.17	●
Fedewa and Ahn, 2011 [27]	Overall ES: g = 0.28	●●	Mathematics: g = 0.44, 95% CI [0.27, 0.61]	●●
	Total achievement: g = 0.27, 95% CI [0.19, 0.35]	●●	Reading: g = 0.36, 95% CI [0.14, 0.58]	●●
	Grade point average: g = 0.24, 95% CI [0.11, 0.38]	●●	Language: g = 0.22, 95% CI [0.10, 0.34]	●●
			Science: g = 0.15, 95% CI [−0.04, 0.34]	⊘
Martin et al., 2014 [28]	Overall ES: SMD = 0.19, 95% CI [−0.36, 0.75]	⊘	Mathematics: SMD = 17.94, 95% CI [−18.44, 54.32]	⊘
			Reading: SMD = 0.07, 95% CI [−2.14, 2.28]	⊘
			Language: SMD = 27.97, 95% CI [−5.35, 61.29]	⊘
Spruit et al., 2016 [30]	Overall ES: mean d = 0.367, 95% CI [0.038, 0.697]	●●	N*/*R	
Álvarez-Bueno et al., 2017 [31]	N*/*R		Mathematics: d = 0.21, 95% CI [0.09, 0.33]	●●
			Reading d = 0.13, 95% CI [0.02, 0.24]	●
			Language: d = 0.16, 95% CI [−0.06, 0.37]	⊘
Watson et al., 2017 [32]	Overall ES: SMD = 0.28, 95% CI [−0.18, 0.73]	⊘	N*/*R	
	Progress monitoring: SMD = 1.03, 95% CI [0.22, 1.84]	●●●		
	Standardised test: SMD = −0.13, 95% CI [−0.72, 0.46]	⊘		
de Greeff et al., 2018 [33]	Overall ES for acute PA: g = 0.09, 95% CI [−0.05, 0.22]	⊘	Mathematics, acute PA: g = −0.18, 95% CI [−0.48, 0.13]	⊘
	Overall ES for chronic PA: g = 0.26, 95% CI [0.02, 0.49]	●●	Mathematics, chronic PA: g = 0.09, 95% CI [−0.17,0.35]	⊘
			Reading, acute PA: g = 0.17, 95% CI [−0.08, 0.41]	⊘
			Reading, chronic PA: g = 0.15, 95% CI [−0.15, 0.46]	⊘
			Spelling, acute PA: g = 0.25, 95% CI [0.03, 0.48]	●●
			Spelling, chronic PA: g = 0.34, 95% CI [−0.23, 0.92]	⊘
Martin et al., 2018 [34]	N*/*R		Mathematics: SMD = 0.49, 95% CI [−0.04, 1.01]	⊘
			Reading: SMD = 0.10, 95% CI [−0.30, 0.49]	⊘
Bedard et al., 2019 [35]	Overall ES: SMD = 0.28, 95% CI [0.09, 0.47]	●●	Mathematics: SMD = 0.08, 95% CI [−0.09, 0.26]	⊘
	ES for primary school: SMD = 0.21, 95% CI [0.009, 0.340]	●●	Reading: SMD = 0.04, 95% CI [−0.16, 0.24]	⊘
	ES for middle school: SMD = −0.01, 95% CI [−0.52, 0.50]	⊘	Language: SMD = 1.07, 95% CI [0.42, 1.72]	●●●
			Spelling: SMD = 0.19, 95% CI [−0.02, 0.40]	⊘
			Science: SMD = 0.57, 95% CI [−0.46, 1.61]	⊘
			Geography: SMD = 0.99, 95% CI [0.02, 1.96]	●●●
Phansikar et al., 2019 [24]	N*/*R		Mathematics: pooled OR = 1.18, 95% CI [0.40, 3.48]	⊘
Ruiz-Hermosa et al., 2019 [23]	N/R		Mathematics: d = −0.33, 95% CI [−0.83, 0.17]	⊘
			Language: d = −0.37, 95% CI [−0.88, 0.15]	⊘
Sneck et al., 2019 [36]	N/R		Mathematics: d = 0.23	●●
Dudley and Burden, 2020 [37]	Overall ES: d = 0.14	●	N*/*R	

CI, Confidence Interval; d, Cohen’s d; g, Hedges’ g; ES, Effect Size; N/R, Not Reported; OR, Odds Ratio; PA, Physical Activity; SMD, Standardised Mean Differences. Effects: ● beneficial; ⊘ null; ● Small effect size; ●● medium effect size; ●●● large effect size.

## Appendix A. Full Search Strategy

### Appendix A.1. PubMed

(“physical activity” [Title/Abstract] OR exercise [Title/Abstract] OR “physical education” [Title/Abstract] OR “active transport” [Title/Abstract] OR “active mobility” [Title/Abstract] OR “active commuting” [Title/Abstract] OR “active travel” [Title/Abstract] OR walking [Title/Abstract] OR cycling [Title/Abstract] OR running [Title/Abstract] OR training [Title/Abstract] OR sport [Title/Abstract]) AND (academic [Title/Abstract] OR school [Title/Abstract]) AND (achievement [Title/Abstract] OR performance [Title/Abstract] OR attainment [Title/Abstract] OR function [Title/Abstract] OR result [Title/Abstract] OR cognitive [Title/Abstract] OR cognition [Title/Abstract]) AND (child* [Title/Abstract] OR school-age [Title/Abstract] OR schoolchildren [Title/Abstract] OR adolescents [Title/Abstract] OR youth [Title/Abstract]) AND (“systematic review” [Title/Abstract] OR meta-analysis [Title/Abstract]).

### Appendix A.2. Cochrane Database of Systematic Reviews

“physical activity” OR exercise OR “physical education” OR “active transport” OR “active mobility” OR “active commuting” OR “active travel” OR walking OR cycling OR running OR training OR sport (Title Abstract Keyword) AND academic OR school (Title Abstract Keyword) AND achievement OR performance OR attainment OR function OR result OR cognitive OR cognition (Title Abstract Keyword) AND child* OR school-age OR schoolchildren OR adolescents OR youth (Title Abstract Keyword) AND “systematic review” OR meta-analysis (Title Abstract Keyword).

### Appendix A.3. Web of Science

“physical activity” OR exercise OR “physical education” OR “active transport” OR “active mobility” OR “active commuting” OR “active travel” OR walking OR cycling OR running OR training OR sport [Title/Abstract/Keywords] AND academic OR school [Title/Abstract/Keywords] AND achievement OR performance OR attainment OR function OR result OR cognitive OR cognition [Title/Abstract/Keywords] AND child* OR school-age OR schoolchildren OR adolescents OR youth [Title/Abstract/Keywords] AND “systematic review” OR meta-analysis [Title/Abstract/Keywords].

### Appendix A.4. Scopus

(“physical activity” OR exercise OR “physical education” OR “active transport” OR “active mobility” OR “active commuting” OR “active travel” OR walking OR cycling OR running OR training OR sport) [Title/Abstract/Keywords] AND (academic OR school) [Title/Abstract/Keywords] AND (achievement OR performance OR attainment OR function OR result OR cognitive OR cognition) [Title/Abstract/Keywords] AND (child* OR school-age OR schoolchildren OR adolescents OR youth) [Title/Abstract/Keywords] AND (“systematic review” OR meta-analysis) [Title/Abstract/Keywords].

### Appendix A.5. LILACS

(“physical activity” OR exercise OR “physical education” OR “active transport” OR “active mobility” OR “active commuting” OR “active travel” OR walking OR cycling OR running OR training OR sport) [Title/Abstract/Subject] AND (academic OR school) [Title/Abstract/Subject] AND (achievement OR performance OR attainment OR function OR result OR cognitive OR cognition) [Title/Abstract/Subject] AND (child* OR school-age OR schoolchildren OR adolescents OR youth) [Title/Abstract/Subject] AND (“systematic review” OR meta-analysis) [Title/Abstract/Subject].

## Appendix B. Assessment of Methodological Quality, Using AMSTAR 2 Tool

**Table A1:** Assessment of the methodological quality using AMSTAR 2 tool.

Author, Year	Item															
	1	2	3	4	5	6	7	8	9	10	11	12	13	14	15	16
Sibley and Etnier, 2003	No	No	No	Partial Yes	No	No	No	No	No	No	Yes	No	No	No	No	No
Strong et al., 2005	No	No	No	No	No	No	No	No	No	No	No meta-analysis conducted	No meta-analysis conducted	No	No	No meta-analysis conducted	No
Murray et al., 2007	No	No	Yes	No	No	Yes	No	No	No	No	No meta-analysis conducted	No meta-analysis conducted	No	No	No meta-analysis conducted	No
Trudeau and Shephard, 2008	No	No	No	No	No	No	No	Partial Yes	No	No	No meta-analysis conducted	No meta-analysis conducted	No	No	No meta-analysis conducted	Yes
Fedewa and Ahn, 2011	No	No	No	Partial Yes	No	No	Partial Yes	Partial Yes	No	No	Yes	No	No	No	Yes	Yes
Rasberry et al., 2011	No	No	Yes	Partial Yes	Yes	Yes	No	No	No	No	No meta-analysis conducted	No meta-analysis conducted	No	No	No meta-analysis conducted	No
Singh et al., 2012	No	No	No	Partial Yes	No	No	No	No	Yes	No	No meta-analysis conducted	No meta-analysis conducted	Yes	Yes	No meta-analysis conducted	Yes
Haapala, 2012	No	No	No	Partial Yes	No	No	Partial Yes	Partial Yes	Yes	No	No meta-analysis conducted	No meta-analysis conducted	No	No	No meta-analysis conducted	No
Lees and Hopkins, 2013	No	No	Yes	Partial Yes	No	No	Partial Yes	No	Partial Yes	No	No meta-analysis conducted	No meta-analysis conducted	No	Yes	No meta-analysis conducted	No
Pucher, Boot and de Vries, 2013	No	No	No	Partial Yes	Yes	No	Partial Yes	Yes	No	No	No meta-analysis conducted	No meta-analysis conducted	No	Yes	No meta-analysis conducted	No
Busch et al., 2014	No	No	No	Partial Yes	Yes	Yes	Partial Yes	Partial Yes	Yes	No	No meta-analysis conducted	No meta-analysis conducted	Yes	No	No meta-analysis conducted	No
Martin et al., 2014	Yes	Yes	Yes	Yes	Yes	Yes	Yes	Yes	Yes	Yes	Yes	Yes	Yes	Yes	No	Yes
Conde and Tercedor, 2015	No	No	No	Partial Yes	No	No	Partial Yes	Partial Yes	No	No	No meta-analysis conducted	No meta-analysis conducted	No	No	No meta-analysis conducted	No
Esteban-Cornejo et al., 2015	No	No	No	Partial Yes	No	No	Partial Yes	No	No	No	No meta-analysis conducted	No meta-analysis conducted	No	No	No meta-analysis conducted	Yes
Ferreira-Vorkapic et al., 2015	No	No	No	Partial Yes	Yes	Yes	Yes	Partial Yes	No	No	Yes	No	No	No	No	Yes
Mura et al., 2015	No	No	No	Partial Yes	No	No	No	Yes	No	No	No meta-analysis conducted	No meta-analysis conducted	No	No	No meta-analysis conducted	No
Norris et al., 2015	No	No	No	No	No	No	No	Partial Yes	Yes	No	No meta-analysis conducted	No meta-analysis conducted	No	No	No meta-analysis conducted	Yes
Donnelly et al., 2016	No	No	No	Partial Yes	Yes	No	Partial Yes	Yes	Yes	No	No meta-analysis conducted	No meta-analysis conducted	Yes	Yes	No meta-analysis conducted	Yes
Poitras et al., 2016	Yes	Yes	No	Partial Yes	Yes	No	Partial Yes	Partial Yes	Yes	No	No meta-analysis conducted	No meta-analysis conducted	Yes	Yes	No meta-analysis conducted	Yes
Spruit et al., 2016	Yes	No	Yes	Partial Yes	Yes	Yes	Yes	Partial Yes	No	No	Yes	No	No	Yes	Yes	No
Álvarez-Bueno et al., 2017	No	Yes	No	Partial Yes	Yes	Yes	Yes	Yes	Yes	No	Yes	Yes	No	Yes	Yes	Yes
Li et al., 2017	No	No	No	Partial Yes	Yes	Yes	Partial Yes	Yes	Yes	No	No meta-analysis conducted	No meta-analysis conducted	Yes	Yes	No meta-analysis conducted	No
Marques et al., 2017	No	No	No	Partial Yes	No	No	Partial Yes	Yes	Yes	No	No meta-analysis conducted	No meta-analysis conducted	Yes	Yes	No meta-analysis conducted	No
Martin and Murtagh, 2017	No	No	No	Partial Yes	Yes	No	Partial Yes	Yes	Yes	No	No meta-analysis conducted	No meta-analysis conducted	Yes	Yes	No meta-analysis conducted	No
Ruiz-Ariza et al., 2017	No	No	No	Partial Yes	Yes	No	Partial Yes	Partial Yes	No	No	No meta-analysis conducted	No meta-analysis conducted	No	No	No meta-analysis conducted	Yes
Schneider, Pharr and Bungum, 2017	No	No	No	Partial Yes	Yes	No	Partial Yes	Partial Yes	No	No	No meta-analysis conducted	No meta-analysis conducted	No	No	No meta-analysis conducted	No
Watson et al., 2017	No	Yes	No	Partial Yes	Yes	No	Yes	Partial Yes	Yes	No	Yes	Yes	Yes	Yes	No	Yes
Marques et al., 2017	No	Yes	No	Yes	Yes	Yes	Partial Yes	No	Yes	No	No meta-analysis conducted	No meta-analysis conducted	Yes	Yes	No meta-analysis conducted	Yes
Daly-Smith et al., 2018	No	Yes	Yes	Partial Yes	Yes	Yes	Partial Yes	Yes	Yes	No	No meta-analysis conducted	No meta-analysis conducted	Yes	Yes	No meta-analysis conducted	Yes
de Greeff et al., 2018	Yes	No	Yes	Partial Yes	Yes	No	Yes	Partial Yes	Yes	No	Yes	No	No	Yes	Yes	Yes
Martin et al., 2018	Yes	Yes	Yes	Yes	Yes	Yes	Yes	Yes	Yes	Yes	Yes	Yes	Yes	Yes	No	Yes
Bedard et al., 2019	Yes	No	Yes	Partial Yes	Yes	Yes	Yes	Partial Yes	Yes	No	Yes	Yes	Yes	Yes	Yes	Yes
Gunnell et al., 2019	Yes	Yes	No	Partial Yes	Yes	Yes	Yes	Yes	Yes	No	No meta-analysis conducted	No meta-analysis conducted	Yes	Yes	No meta-analysis conducted	Yes
Phansikar et al., 2019	No	No	No	Partial Yes	Yes	No	Yes	Partial Yes	Yes	No	Yes	No	Yes	Yes	No	Yes
Ruiz-Hermosa et al., 2019	No	Yes	Yes	Partial Yes	Yes	Yes	Yes	Partial Yes	Yes	No	Yes	Yes	Yes	Yes	No	Yes
Singh et al., 2019	No	Yes	Yes	Partial Yes	Yes	Yes	No	Partial Yes	Yes	No	No meta-analysis conducted	No meta-analysis conducted	Yes	Yes	No meta-analysis conducted	Yes
Sneck et al., 2019	Yes	No	Yes	Partial Yes	Yes	Yes	Yes	Partial Yes	Yes	No	Yes	Yes	No	Yes	No	Yes
Chacón-Cuberos et al., 2020	No	No	Yes	Partial Yes	No	No	No	No	No	No	No meta-analysis conducted	No meta-analysis conducted	No	Yes	No meta-analysis conducted	No
Dudley and Burden, 2020	No	No	No	Partial Yes	Yes	Yes	Yes	Partial Yes	Yes	No	Yes	No	No	No	Yes	Yes
Masini et al., 2020	Yes	Yes	No	Partial Yes	Yes	No	Yes	Partial Yes	Yes	No	Yes	Yes	Yes	Yes	Yes	Yes
Vetter et al., 2020	No	No	No	Partial Yes	Yes	Yes	Yes	Yes	Yes	No	No meta-analysis conducted	No meta-analysis conducted	Yes	Yes	No meta-analysis conducted	No
